# Gender Differences in Determinants and Consequences of Health and Illness

**Published:** 2007-03

**Authors:** Carol Vlassoff

**Affiliations:** Department of Epidemiology and Community Medicine, University of Ottawa, Canada

**Keywords:** Gender identity, Health, Tropical medicine, Developing countries, Developed countries, Chronic disease, Impact studies, Review literature

## Abstract

This paper uses a framework developed for gender and tropical diseases for the analysis of non-communicable diseases and conditions in developing and industrialized countries. The framework illustrates that gender interacts with the social, economic and biological determinants and consequences of tropical diseases to create different health outcomes for males and females. Whereas the framework was previously limited to developing countries where tropical infectious diseases are more prevalent, the present paper demonstrates that gender has an important effect on the determinants and consequences of health and illness in industrialized countries as well. This paper reviews a large number of studies on the interaction between gender and the determinants and consequences of chronic diseases and shows how these interactions result in different approaches to prevention, treatment, and coping with illness. Specific examples of chronic diseases are discussed in each section with respect to both developing and industrialized countries.

## INTRODUCTION

Gender refers to “the array of socially constructed roles and relationships, personality traits, attitudes, behaviours, values, relative power and influence that society ascribes to the two sexes on a differential basis. Gender is relational—gender roles and characteristics do not exist in isolation, but are defined in relation to one another and through the relationships between women and men, girls and boys” (1). Simply put, sex refers to biological differences, whereas gender refers to social differences.

In the last decade, a considerable amount of research has been conducted in the area of gender and health, including gender differences in vulnerability to, and the impact of, specific health conditions. Gender has been shown to influence how health policies are conceived and implemented, how biomedical and contraceptive technologies are developed, and how the health system responds to male and female clients (2).

Gender analysis in health has been undertaken mainly by social scientists who observed that biological differences alone cannot adequately explain health behaviour. Health outcomes also depend upon social and economic factors that, in turn, are influenced by cultural and political conditions in society. To understand health and illness, both sex and gender must be taken into account.

This paper builds upon a gender framework from the field of tropical diseases (3) by examining to what extent the framework applies equally to non-communicable diseases. The framework includes the social, economic and personal/biological determinants and consequences of tropical diseases and analyzes how gender interacts with these factors to produce different outcomes for males and females. For example, the gender differences in the social determinants of tropical diseases include the different roles of men and women in the household, status within the household and community, and cultural norms affecting risks of infection. These factors influence exposure of women and men to diseases such as malaria because men are more likely to be exposed to mosquitoes in certain work environments such as forestry or mining (3). The gender differences in the consequences of tropical diseases include how illness is experienced, treatment-seeking behaviour, nature of treatment, and care and support received from the family and care providers. In the case of HIV-associated disease, for instance, the economic consequences may be worse for women who are left with families to support when husbands become infected and die, or they may not be able to earn income or support their families when they themselves are ill.

Whereas this framework previously was limited only to developing countries where tropical diseases are mainly found, this paper expands the analysis to include industrialized countries as well. The paper brings together the findings of various studies to identify how gender interacts with the determinants and consequences of health and illness. Whereas previous research based on this framework was limited to developing countries, the present analysis demonstrates that gender has an important effect on the determinants and consequences of non-communicable diseases and conditions in both developing and industrialized countries. In each section of the paper, one example of a chronic disease or condition is provided to illustrate how the gender framework can be equally applied to developing and industrialized societies.

There is no systematic body of knowledge on gender and chronic diseases, although there is a considerable literature emerging on specific diseases such as those discussed in this paper. Based on research findings on gender, several hypotheses have been proposed. Verbrugge, for example, argued that gender differences are more pronounced for prolonged, mild conditions than for acute, life-threatening or severe ones (4). However, further research on specific diseases, including tropical infectious diseases, has added new findings that need to be taken into account. Charmaz notes the importance of examining gender differences in non-communicable diseases and that the experience of illness is strongly related to gender identities (5).

The following analysis, therefore, brings together two areas of investigation—tropical infectious diseases and chronic non-communicable diseases—by showing that the framework from tropical diseases also applies to chronic diseases. It also draws out conclusions regarding gender and chronic diseases by comparing the results of the various studies of different diseases or conditions.

## MATERIALS AND METHODS

This paper is based on a review of published articles in the area of gender and health. By way of illustration, examples of non-communicable diseases or conditions are highlighted under the headings of social, economic and biological determinants and consequences respectively to demonstrate their interaction with gender variables. The examples are not related to one another but have been selected because they have been studied in both developing- and industrialized-country context and because they demonstrate the interaction of gender variables with social, economic and biological factors and how these produce different outcomes for males and females.

## RESULTS

### Gender differences in determinants of health and illness

This section reviews evidence of gender differences in the social, economic and biological determinants of health and illness, focusing on three non-communic-able diseases or conditions: nutrition for social determinants, mental illness for economic determinants, and longevity for biological determinants.

#### Gender differences in social determinants of health and illness

Social factors, such as the degree to which women are excluded from schooling, or from participation in public life, affect their knowledge about health problems and how to prevent and treat them. The subordination of women by men, a phenomenon found in most countries, results in a distinction between roles of men and women and their separate assignment to domestic and public spheres. The degree of this subordination varies by country and geographical or cultural patterns within countries, however, in developing areas, it is most pronounced. In this section, the example of nutrition will demonstrate how gender has an important influence on the social determinants of food-consumption patterns and hence on health outcomes.

Several studies have shown the positive relationship among education of mothers, household autonomy, and the nutritional status of their children (6, 7). During the first 10 years of life, the energy and nutrient needs of girls and boys are the same. Yet, in some countries, especially in South Asia, men and boys often receive greater quantities of higher quality, nutritious food such as dairy products, because they will become the breadwinners (7–15). Das Gupta argued that depriving female children of food was an explicit strategy used by parents to achieve a small family size and desired composition (13). Studies from Latin America also found evidence of gender bias in food allocation in childhood (16–18) and, correspondingly, in healthcare allocation (19).

In developing countries, most studies show preferential food allocation to males over females. Nonetheless, some studies have found no sex differences in the nutritional status of girls and boys (20–22), and others have described differences only at certain times of the life-cycle. For example, research in rural Mexico found no nutritional differences between girls and boys in infancy or preschool, but school-going girls consumed less energy than boys. This was explained by the fact that girls are engaged in less physical activity as a result of culturally-prescribed sex roles rather than by sex bias in food allocation (23).

Studies from developing countries of gender differences in nutrition in adulthood argue that household power relations are closely linked to nutritional outcomes. In Zimbabwe, for example, when husbands had complete control over all decisions, women had significantly lower nutritional status than men (24). Similarly, female household heads had significantly better nutritional status, suggesting that decision-making power is strongly associated with access to and control over food resources. Access of women to cash-income was a positive determinant of their nutritional status. In rural Haiti, the differences in nutritional status for male and female caregivers were examined for children whose mothers were absent from home during the day. Those who were looked after by males, such as fathers, uncles, or older brothers, had poorer nutritional status than children who were cared for by females, such as grandmothers or sisters (25). Ethnographic research conducted by the authors revealed, however, that, while mothers told the interviewers that the father stayed home with the children, it is probable that the father was, in fact, absent most of the day working and that the children were cared for by the oldest child, sometimes as young as five years of age.

The involvement of both men and women in nutritional information and interventions is key to their successful implementation. Unfortunately, in most developing countries, women are selected for nutritional education because they are responsible for the preparation of meals. However, they often lack access to nutritional food because men generally make decisions about its production and purchase. Similarly, men may not provide nutritional food for their families because they have not received information about nutrition. The participation of both men and women is, therefore, fundamental to changing how decisions about food are made and food-consumption patterns and nutrition families (26). The study in rural Haiti referred to above also found positive outcomes through the formation of men's groups which received information on nutrition, health, and childcare. These men, in turn, were resources for education of the whole community (25).

The gender differences are also found in the social determinants of nutrition in industrialized countries, although their manifestations are different. For example, gender plays an important role in determining risk factors for eating disorders, which influence nutritional outcomes. The most common of these are anorexia nervosa, bulimia nervosa, and binge eating (BED) (27–28). The root causes are only partly understood. Biomedical and psychological theories include hormonal imbalance, malfunctioning of serotonin in the brain, genetic explanations, and emotional problems expressed by abnormal relationships with food. Sociocultural explanations include the emphasis placed on the ‘ideal’ female body shape in western society. Experts agree that a key factor is the desire to please others. These characteristics are linked to ‘negative femininity’—behaviours associated with passivity, dependence, unassertiveness, and low self-esteem. Dieting and bingeing may be used for improving body image and self-esteem. Concern with body image is particularly strong in adolescence where the differences in calcium intake and a more sedentary lifestyle are pronounced (29). Results of a study of 1,755 adolescents in the United States also showed that, during adolescence, intake of fruits and vegetables was generally low for both boys and girls and that their consumption was related to consciousness about controlling their weight (30). Among men, dieting and bingeing seem to be more common among gay men and sports competitors than in heterosexuals (31).

Many studies have demonstrated the effect of social support on nutrition in older adults, with a positive impact being seen among those who are married, especially men (32–34). This has been explained by several factors—the greater likelihood to skip meals when living alone, or to eat filling but unhealthy products and snacks. Women who are alone may not be able to afford an adequate diet, or they may be less motivated to cook for themselves when they are accustomed to providing for others (35–36).

The gender differences in nutritional risk were studied among an older sample of black and white community dwelling residents in Alabama, USA (37). The study took into account social support, social isolation, and social capital as possible determinants of nutritional risk. Social capital was defined to include neighbourhoods, trust people felt in their security, and religion. The study found important gender and racial differences between different groups, black men being the most affected by poor nutrition if lacking in social support and capital. White men were in the best overall position, with white women in the second best position, and black women in the third. The study found that social isolation and lower income contributed most to nutritional risk for all groups, except black men, for whom lack of social support and capital were the most important determinants of nutritional risk.

The studies discussed in this section demonstrate that gender matters in terms of nutritional outcomes, but, at the same time, generalizations as to how gender affects the social determinants of nutrition can be misleading. The complexity of social, economic and cultural contexts and also demographic and epidemiological indicators must be taken into account to fully understand the additional impact that gender has.

### Gender differences in economic determinants of health and illness

Productive labour is usually defined as labour performed outside the household in income-generating employment; reproductive labour includes work done within the household, such as food preparation, childcare, housework, care of livestock and kitchen gardens. Reproductive labour, in addition to reproducing the daily conditions of domestic survival, also assures the reproduction of human values, attitudes, and culture. In both industrialized and developing countries, women spend considerably more time than men in reproductive, volunteer and other unpaid labour, whereas men spend significantly more time in productive, remunerated work (3).

In most cultures, productive and reproductive activities are valued differently. Generally, earning an income brings greater autonomy, decision-making power, and respect in society. Given the greater involvement of men in the paid labour force and their higher earnings even when domestic and other activities of women are costed, they generally enjoy more autonomy and higher social status. The gender differences in economic status and purchasing power affect the health-seeking behaviour and health outcomes of men and women. Recent schools of thought have recognized that many types of non-market or reproductive labour are also productive. For example, gender-aware economics includes unpaid caring work in the home in the concept of productive labour and informal paid work, such as home-based income-generating activities and work in non-profit or non-governmental organizations.

Research on gender and the economic determinants of health and illness is relatively scarce, especially in the area of non-communicable diseases. The example of mental health is used here because there is considerable research on this topic in industrialized countries, and some studies can also be cited from developing countries. The relative paucity of research on gender and economic aspects of mental health in developing countries reflects the fact that mental health services are less numerous and comprehensive than those in industrialized countries. Nonetheless, interesting studies have been carried out in several countries that demonstrate a clear relationship between economic factors and mental health by gender.

A study of gender and mental health in China that combined historical, epidemiological and qualitative data found significantly higher rates of schizophrenia among women than among men, a finding contrary to western studies in which men suffer more from schizophrenia (38). Interestingly, however, men occupied more hospital beds than women in psychiatric hospitals, in which at least three-quarters of patients were suffering from schizophrenia, indicating that hospital-bed occupancy did not reflect the male-female ratio of people affected by the disease. While several possible reasons for this imbalance were cited, significant gender differences in ability to pay were noted. Men were much more likely to have health insurance from their employers than women, who tended to be treated more as charity cases. Reports from other parts of the world show that women constitute the large majority of individuals seeking psychological services (39). Given this gender imbalance, services are not positioned to respond adequately to their female clients (40).

The gender differences in the economic determinants of mental health were also encountered in South Korea. A recent study examined the impact on men and women of escalating job insecurity due to increasing numbers of non-standard workers. The proportion of non-standard workers was considerably higher among women than among men. In general, non-standard workers (part-time, temporary, and daily labour) were more likely to suffer from mental problems than standard employees, and non-standard female workers suffered more mental illness than men, in terms of self-reported depression and suicidal thoughts (41). Married women reported more psychological problems than single women, and the pattern was reversed for men.

The links among mental health, gender, and economic status were clear in several aspects of the Korean study. Women had about twice the incidence of poor mental health indicators than men, and the mental health problems increased as income declined. This is also true of other studies (42–44). The reasons within the Korean context were explained by Kim *et al.* (41) by the fact that, even among non-standard workers, men tended to occupy higher-level positions in construction and manufacturing, whereas women were employed more in unskilled jobs. The average wage for women was less than 40% of that of men, and only a tenth of women received fringe benefits. Women also had many other family responsibilities which they had to fulfill, in addition to their paid labour.

Results of research in industrialized countries consistently indicate that women have higher rates of anxiety and depression than men, independently of race, time, age, and rural-urban residence. The fact that men have greater control over resources, and decision-making power is one explanation, but there is considerable evidence that even when women have control over resources and income through employment anxiety and depression is not necessarily reduced (45). A national cross-sectional survey of British adults found that people in the most disadvantaged socioeconomic positions reported higher rates of affective disorders and minor physical illnesses than those in higher positions. The gender differences were found in the other socioeconomic classes. Among healthy older women, for example, those in the skilled occupational class reported the highest rates of affective disorders, whereas among men, the highest rates were found in the clerical class. Generally, in positions occupied by both the sexes, and among men and women with similar income levels, women reported higher rates of both affective disorders and minor physical morbidity (46). The authors concluded that the experience of a particular social or occupational position might be different for men and women, explaining why women consistently experience more affective disorders and minor physical morbidity.

In an analysis of gender, employment, and mental health, Rosenfield compared men and women from the United States using measures of power in work and family, demands on time and personal control, and symptoms of depression and anxiety (45). Men and women with similar demands on their time in family and work situations had similar symptoms of psychological distress. However, women in situations of higher demands, either as unemployed housewives or as working women with significant familial responsibilities, had higher rates of depression and anxiety than men. Thus, the gender differences in economic roles strongly influence mental health outcomes.

### Gender differences in biological determinants of health and illness

The gender differences in the biological determinants of health and illness include differential genetic vulnerability to illness, reproductive and hormonal factors, and differences in physiological characteristics during the life-cycle. Until recently, a male model of health was used almost exclusively for clinical research, and the findings were generalized to women, except for the reproductive period. Clinical trials typically excluded women to protect them and their unborn children from possible negative effects. However, research in the United States in the early 1990s seriously questioned the validity of a male model for female health issues and highlighted significant gender differences in the biological determinants of health and illness (47). For example, protocols for the diagnosis and treatment of heart disease, the number one cause of all deaths in the United States, were based upon findings from middle-aged white male patients. As a result, women were diagnosed later with more advanced disease and were consequently harder to treat successfully.

Questions about gender differences in heart disease, mental illness, and osteoporosis led to the important recommendation that women be included in clinical studies to uncover gender differences and their impact on the prevention, diagnosis, and treatment of disease. In 1993, the U.S. Food and Drug Administration established an Office of Women's Health and published “Guidelines for the Study and Evaluation of Gender differences in the Clinical Evaluation of Drugs” which ended the policy of exclusion, recommending that women be appropriately represented in clinical studies and that their findings be analyzed from a gender perspective (48). Such policies are still not implemented in most of the developing world.

The interaction between biological and social determinants is also important when considering gender differences in health. The biological differences can be amplified or suppressed by socialization and how society responds to sex-specific behaviour. Social norms endorsing particular kinds of behaviour may exacerbate negative tendencies, such as violence, or reinforce positive propensities, such as nurturing. By contrast, socialization can suppress innate negative or positive tendencies.

The example of longevity is used here for demonstrating how gender affects the biological determinants of health conditions. Universally, women live longer than men but the gender gap is greatest in developed societies where women outlive men by about seven years, on average (49). The most apparent gender difference in the ageing process is women's finite period of reproductive functioning. Their menopausal transition is associated with mood fluctuations and a decline in sexual interest relating to hormonal change. As they age, men and women suffer from similar types of illnesses but men tend to suffer from acute illnesses for relatively short periods before they die (49). Women, by contrast, have a longer life, marked by many chronic non-life-threatening disabilities that can greatly affect the quality of their lives. For example, osteoporosis, due to a natural decline in bone density after menopause, affects mainly women (50).

There has been considerably more research on gender and longevity in industrialized countries than in developing countries. Senenayake points out that health policies seem to assume that men and women's problems converge after menopause (49), whereas, in fact, they continue to be distinct. She notes, for example, that there is a lack of sex-specific data for elderly people in developing countries. However, the available data reported from developing countries indicate similar gender differences worldwide: women have significantly higher rates of arthritis, osteoporosis, diabetes, and hypertension than men (51). In a study of adult diseases in Bangladesh, Malaysia, Jamaica, and the United States, Strauss et al. found that women reported more health problems than men, regardless of economic status (52).

Older women in both developing and industrialized countries are more likely to live alone than men (53). As noted above, isolation can severely affect the health of older people, and given the lower economic status of women, they are less likely to be able to seek help (50). A study of elderly males and females in Egypt, for example, found that females who had lived all their lives in rural areas and were living in a fair or poor residence were more likely to be disabled than women in better circumstances (54). For Egyptian males, only illiteracy was associated with disablement. This was attributed to the fact that literacy is much more prevalent among men, and those who are illiterate are, therefore, more likely to be poor. For women, living in rural areas is associated with having large families and a tendency to rely on traditional healers for births and medical needs. They are, thus, more exposed than men to poor medical care for reproductive healthcare and, consequently, more at risk of infection. They also have less access to medicines to treat morbid conditions. For women but not for men, living alone was associated with increased odds of disablement (54).

An interesting study by Rahman found important gender differences in elderly mortality in Bangladesh (55). In a longitudinal study in the Matlab surveillance area, a large sample of men and women aged 60 years and older were followed for eight years to determine the impact of several social, economic and demographic variables. The study found that household heads, whether male and female, had lower mortality, and the presence of a partner had a significant positive impact on men, but a positive impact on women only when their husbands were the heads of the household. Moreover, the presence of an adult son was correlated with lower mortality among women but not men. These findings indicate that individual access, to resources as opposed to joint access, is an important determinant in the survival of elderly people (56).

In industrialized countries, the impact of gender on the biological determinants of longevity is also evident, for example, in the quality of life of elderly people. In a study of 14,000 men and women aged 60 years and above living in their homes in Britain, the gender differences were found in living arrangements for people living with severe disabilities (56). Half of these older women lived alone compared to one-quarter of older men. Most men with severe or moderate disability lived with their spouse and received care from them, whereas most women lived alone and had to rely on help from outsiders.

The British study examined self-assessed health to test the validity of the common assumption that women over-report morbidity (57). There was a little gender difference in self-assessed morbidity, once class, income, age, and level of functional disability were taken into account. In fact, results of multivariate analysis indicated that, when the greater functional disability of older women was included, older women reported less poor health than older men. These findings illustrate the importance of re-examining common gender-based assumptions and of assuring that comparisons between men and women are based on similar socioeconomic and demographic groups. Moreover, gender relations and their impact on biological factors are changing, as women increasingly assume positions traditionally occupied by men and vice versa.

Several studies of the ‘will to live’ have found that women have a weaker desire to prolong life than men, in terms of refusing life-sustaining care (57–58) or a wish to die sooner if terminally ill (59–60). In a study of gender differences among men and women aged 70 years or older in Israel, the will to live was found to be affected by the state of health of the elderly, those in poor health more likely to show the gender differences referred to above (59). As in other studies, living with a partner was a significant predictor of the will to live among men, but not among women (61–62).

### Gender differences in consequences of health and illness

This section reviews research on how gender affects the social, economic and biological consequences of health and illness, focusing on three non-communicable diseases or conditions: diabetes for social consequences, domestic violence for economic consequences, and occupational health for biological consequences.

#### Gender differences in social consequences of health and illness

The gender differences in the social consequences of health and illness include how illness affects men and women, including health-seeking behaviour, the availability of support networks, and the stigma associated with illness and disease. Men and women respond differently when ill, in terms of time before acknowledging that they are ill, recovery time, and how women and men are treated by their families and society.

In developing countries, men seek treatment more frequently at formal health services, whereas women are more likely to self-treat or use alternative therapies. This has been explained by factors, such as multiple roles of women which limit their activities mainly to the domestic sphere and make it difficult for them to go to clinics during opening hours. By contrast, traditional healers or community shops are easier to access and will often accept delayed payment or payment in kind or delayed. Traditional healers also provide explanations in ways that are easily understood, in contrast to the more scientific explanations of clinic staff (3). Women are often treated in an inferior way at health services and are blamed for coming late or for not bringing their children for regular immunization or check-ups. This only exacerbates women's reluctance to access healthcare, even when other access barriers are removed (63). Insensitive treatment by health personnel is also a problem in industrialized countries, although in these situations women have more options for restitution.

The lower social status of women influences how society responds when they are affected by stigmatizing illnesses, such as HIV/AIDS, leprosy, tuberculosis, and mental illness. While both men and women suffer considerable discrimination and from society, women are more marginalized by these health problems.

The example of diabetes, a non-communicable disease, demonstrates the gender differences in its social consequences. Research on gender differences in the social consequences of diabetes is limited, especially in developing countries. Even in industrialized countries, the studies in this area are difficult to compare because they deal with different variables, measurement tools, and outcomes. However, it is possible to draw some conclusions from the existing literature which are relevant from a gender perspective.

A recent study from Trinidad found that men with type 2 diabetes mellitus were less compliant with treatment than women and that they were less satisfied with the way they were treated in the dispensary and clinic they attended. Men tended to smoke and drink alcohol much more frequently and, hence, were predisposed to a wider range of health risks, including hypertension and cerebrovascular and cardiovascular diseases. The authors hypothesized that men with diabetes probably had a lower life expectancy than women (64).

Some evidence from India showed that boys have better access to care for insulin-dependent diabetes mellitus (IDDM) than girls. The reasons were not studied but it is likely that son preference plays a role here, in keeping with other health-related research findings. Sridhar noted that mothers tended to take responsibility for looking after diabetic children, which could result in alienating fathers and making them uninterested in helping to care for their children (65).

A common finding in developing countries is that urban dwellers have a higher prevalence of diabetes than rural residents because of the shift from low to high fat intake (66–68) but there does not seem to be a consistent pattern of gender differences within this rural-urban categorization.

Studies on gender differences in diabetes in industrialized countries have focused on how men and women or girls and boys cope with the illness, including the types of coping strategies they develop. Perhaps the most common finding is that women and girls generally have a more negative way of dealing with diabetes than men and boys. Anxiety and depression are more common among females (69–71). In a sample of adolescents in the United States, even after controlling for other correlates, such as levels of knowledge about diabetes and metabolic control, girls were less positive about their illness than boys (71).

More research is needed to understand the link between diabetes and depression (71). Some researchers have reported a higher incidence of eating disorders among diabetic adolescent girls than among boys with IDDM or than among non-diabetic girls (72). Another possible explanation is that girls may internalize stress more than boys who tend to deal with their stress by more positive behaviour, such as practising sports and following a controlled diet. Cruickshanks reported that adolescent diabetic girls are engaged in fewer physical activities than boys, possibly contributing to poorer diabetic control (73).

As has been found in other studies cited in this paper, the assistance and emotional support received from their spouses has been found to influence health outcomes for people with diabetes. A study of men and women with diabetes over a 10-year period found that male patients reported more satisfaction with the support received than women (74). Similarly, wives of diabetic patients reported fewer problems in giving medication and in testing blood glucose levels than did husbands of female patients. Men with diabetes received more support from their partners than women, as demonstrated by the greater attendance of wives in education programmes than husbands of diabetic women. Men reported better self-care, such as eating meals on time, less binge eating, and less late insulin injections. Men also recounted fewer incidents of ketonuria, better blood sugar levels, and fewer diabetes-related complications. Men generally mentioned greater satisfaction with their diet and their treatment regime. Whereas men were less likely to miss work or activities because of their disease, wives of diabetics reported missing more work because of their husband's condition than husbands of female diabetics (74). This again indicates the greater support received from women by men who are ill than the reverse. Similar results concerning positive coping behaviour of men and support from spouses have also been reported elsewhere (75–77).

Research in Sweden on coping strategies of men and women with type 2 diabetes found that women used more negative coping strategies, including resignation, protest, and isolation, whereas men took a more problem-solving approach (78). Another Swedish study found that men under-estimated diabetes-related problems more than women and worried less about long-term complications. However, they were more concerned about the impact of illness on their personal freedom. Although women were more worried about their health, they were able to find positive aspects in having diabetes. Younger people also had more positive attitudes than older people, although they were more likely to consider that the disease had negatively affected their relationships with others (79).

Research in the United Kingdom found similar results as in the Swedish research. Reporting on in-depth interviews with girls and boys with chronic diseases—type 1 diabetes and asthma—Williams found that youngsters managed their conditions in “gendered ways, with the aim of projecting different, gendered identities” (80:394). The majority of girls adapted to the illness by incorporating it into their social and personal identities. Boys tended not to identify with the illness but rather to find ways of combating it or keeping it at bay. These findings support the observations of Charmaz (5) and Prout (81) who emphasized the stigmatizing impact that chronic illness can have on males at different ages. The greater acceptance by girls of their condition had detrimental consequences in that they had lower expectations of themselves and were also less capable of managing their illness by diet and exercise as well as boys did. Boys tended to use exercise as a means of keeping their blood sugar under control, whereas girls were more likely to give themselves more insulin instead.

### Gender differences in the economic consequences of illness

The gender differences in the economic consequences of illness include how work of men and women is affected by illness, such as availability of substitute labour, opportunity costs of health-related actions, available income, and the impact of economic policies.

When poor women in developing countries are ill, they tend to delay seeking modern treatment until their symptoms are too severe to ignore, meanwhile perhaps visiting a traditional healer or local pharmacy. Thus, they take longer to recover and often return to work before they have completely recuperated (82). When men are ill, others encourage them to seek medical help, and hence they are appropriately diagnosed and treated earlier than women. They also receive greater care from wives and others and are not expected to perform other duties until they are better. Women often substitute for their husbands in agricultural work when they are ill but husbands rarely substitute for their wives, and only essential duties are assumed by other family members. When women recover, they are faced with many pending tasks, in addition to their normal work. Those who own small businesses lose necessary income for daily survival, and many have to use their scarce resources for medicines and other health-related costs (82). The fact that women are often paid less for the same jobs as men also means that they have fewer resources to fall back on when they become ill, and their control over their own earnings is often limited (83).

The impact of economic restructuring policies on access to and use of health services by the poor is an issue of growing concern. Given that women universally occupy a lower socioeconomic status, poor women, especially those in female-headed households, are most adversely affected by economic adjustment policies. Health-sector reforms have sought to strengthen private financing, to decentralize services, and to improve service-delivery through private sources. A central question is whether, in the interests of cost cutting and efficiency, costs will be transferred from the remunerated economy to the unremunerated economy, where women predominate.

In this section, we use the example of violence against women to demonstrate the impact of gender on the economic consequences of illness. The section does not separate the discussion clearly into developing and industrialized countries (although examples of both are cited) because domestic violence is a universal phenomenon found in all cultures and at every level of society, and the effects are similar, although they may vary in intensity and severity. Partner violence is mainly aggression by men against women and children, although men are also victims of domestic violence. In the United States, it is estimated that males constitute only 10–15% of victims (84).

Relatively little information is available on the cumulative economic impact of domestic violence but there is growing evidence that it adversely affects women and children's mental health and opportunities for a productive life. Among low-income groups, poverty may exacerbate marital disagreements, and it may be more difficult for poor women to leave violent situations because they have less access to outside help or accommodation (1). It is well-known that violent partners frequently obstruct efforts of women to seek help for themselves and their children, and poor women have little economic power in such situations. The impact of domestic violence is magnified in areas with fewer material resources (85). There may also be substantial under-reporting among the higher socioeconomic groups.

Victims of domestic violence have more difficulty keeping a job because they often miss work due to physical injury. Many female victims of physical or sexual violence are unable to work because of their injuries, or are stalked or harassed at work. The fear and discomfort entailed affects their ability to perform, and they may also be forced to leave their job when the situation becomes unbearable. Women who resist a harassing male colleague or superior may be dismissed (86).

Children who witness domestic violence exhibit many of the same emotional and behavioural problems as children who have been sexually or physically abused, including poor school performance and somatic health complaints. Those who have been abused are more likely to be abusive as adults. The cycle of poverty, gender violence, poor health, and limited economic opportunities is perpetrated throughout the generations (87).

In developing countries, women who are totally dependent for economic livelihood upon their husbands are particularly affected when they suffer domestic abuse. In a study in Mexico, women at risk of abuse and who lived with their own parents or in an extended family were much more likely to be protected from it than those who lived in a nuclear family situation (88). Those who were unable, for economic reasons, to leave their husbands were the worst-off and least able to escape the situation. Finkler explains that poor Mexican men also suffered a different kind of abuse: being looked down upon in society, their dignity is challenged, and they may try to compensate for their frustration through mistreating those who cannot retaliate.

In India, dowry-related violence, sometimes leading to deaths by murder or suicide, is increasingly being documented. Dowry, a Hindu tradition, was originally a way for parents to share their inheritance with their daughters who were not allowed to inherit property. As Fischbach and Herbert observe, this practice has become a ‘crucial marital transaction’ and a way to ‘get rich quick’ (85). Bridal abuse is a way of putting pressure on her family to give them more of their assets, and when a wife is unable to provide them, she may resort to suicide or be killed. Women rarely seek medical help, mainly because of shame. A study in Bangladesh found, for example, that 66% of women were silent about their experience, mainly because of culturally-endorsed acceptance of violence or fear of stigma and greater harm (89). In Uganda as well, lack of economic autonomy was an important reason that women stayed in abusive relationships, “…many of the women experienced poverty so severe that they had literally no option but to remain with husbands who routinely battered them. Their worth and social acceptance was found in marriage and children, making separation or divorce almost impossible” (90).

Another pervasive form of violence against women is rape. This is considered to be among the most under-reported health problems in the world (85), a crime that can have serious psychological, social and economic consequences. In several parts of the world, women who have been raped may be seen as having brought dishonour on their families. In some countries, rape victims may be beaten, killed, or driven to suicide (85). In these situations, even those who survive are likely to face precarious economic futures, as they may be driven away from their families and be left without social or economic support (85).

### Gender differences in biological consequences of illness

Generally, men are more vulnerable to major life-threatening chronic diseases, including coronary heart disease, cancer, cerebrovascular disease, emphysema, cirrhosis of the liver, kidney disease, and atherosclerosis. Women suffer more from chronic disorders, such as anaemia, thyroid and gall bladder conditions, migraine headaches, arthritis, colitis, and eczema. The biological advantage of women appears to be related to their ability to bear children and the physiological systems that permit pregnancy and child bearing, whereas men's health advantage seems to be due to lower levels of role stress, role conflict, and lower societal demands (91).

Men and women have different responses to drugs for treatment. These gender differences are not only biological: gender plays an important role in determining healthy or unhealthy life styles. As men and women modify their behaviour to reduce or increase certain risks, such as stress relating to high-pressure jobs, their respective vulnerability can change over time and across societies.

The gender differences in the biological consequences of health and illness can be illustrated by the example of occupational health. Until recently, little attention was paid to gender differences in occupational health, and most social science literature focused on differences in exposure to health risks (92). The literature on tropical diseases found significant gender differences in the impact of infectious diseases on men and women because of their differential exposure to vectors, such as mosquitoes or sandflies (82). Social scientists are now investigating the impact of different kinds of work environment on health of men and women but considerably more research is needed to confirm early findings in this regard. Research in industrialized countries has shown that working outside the home is related to improved health for women (93–95) because of increased self-confidence and economic independence. Similarly, among men, employment is associated with increased life expectancy (96–97), and unemployed men are at greater risk of psychological problems and early mortality (97).

In developing countries, however, there is insufficient evidence to conclude that non-domestic labour has a positive impact on women's health. Women may suffer more ill-health because labour conditions are generally much poorer in developing countries, their status is lower than men's, and they often assume the large burden of domestic work, in addition to paid labour (83). Research on factory work in both low- and high-income countries has found that women who are employed in monotonous and repetitive work are likely to develop repetitive strain injuries (83) or to be exposed to carcinogenic substances (98, 99). Men are more often employed in dynamic jobs involving physically strenuous activities, such as construction, with considerable lifting and moving of heavy loads.

Work-related accidents resulting in death seem to be much more common among men in both industrialized and developing countries because men are employed in occupations involving greater danger, such as transportation, construction, mining, and fire-fighting. Men in developing countries are also more at risk of accidents than men in high-income countries because of poorer safety regulations and protective equipment (83).

Because of women's double responsibility for both household and outside work, and their lack of decision-making power and often arduous tasks in the workplace, female managers tend to experience more ‘negative stress’ than men (100). In several studies, Oslin has shown that women at all levels of employment reported more such stress (83). For example, women who had to work more than 10 hours of overtime per week had a higher risk of heart attacks than other women, whereas men who worked the same amount of overtime were at lower risk. Moreover, women's level of negative stress increased at the end of the working day, whereas men's level of negative stress decreased considerably.

## DISCUSSION

This paper has reviewed many studies and health and illness examples as they relate to gender differences using a framework from the field of tropical diseases. Clearly, the framework which links gender to the social, economic and biological determinants and consequences of tropical diseases is applicable to non-infectious diseases and conditions too.

Several conclusions regarding the importance of gender for understanding health and illness can be derived from the studies reviewed in this paper. First, gender clearly plays a role in the determinants and consequences of poor health, and it can no longer be assumed that a male model for health also applies to women. The way in which gender affects these determinants and consequences may vary according to the conditions selected and according to the characteristics of the population studied. However, gender analysis is key to understanding the experience of health and how to intervene to prevent illness.

Perhaps, the most common finding across the different chronic diseases and conditions reviewed is the importance of social support, especially by spouses and other family members, in helping people cope positively with their condition. There was a widespread gender bias towards men in terms of the support received from their families, and this helped them respond better to their illness. Women were less likely to receive support, leading to less positive coping. Women were also more prone to accept their condition as part of themselves, rather than to see it as a challenge to be overcome, as their male counterparts tended to do.

The involvement of both men and women in health education and interventions was shown to be an important determinant of their successful uptake. This demonstrates that gender stereotypes need to be examined critically as they stand in the way of the improvements in health that are known to be effective. For example, it was seen that selecting women for nutritional education because they are responsible for the preparation of meals means that men are generally excluded, yet it is men who are heavily involved in the production, sale, and purchase of food. Similarly, not understanding the dynamics of age, ethnicity and gender can be detrimental to desirable health interventions. This was seen in several examples discussed in the paper.

The framework discussed in this paper separated out social economic and biological determinants and consequences of health and illness to bring an organizing structure to a vast number of individual studies on a range of varying health conditions. However, it must be recognized that these determinants and consequences also interact with one another as seen, for example, in the case of domestic violence. Women who are victims of violence miss more work than other women because of their injuries and hide their injuries from others, including health services, because of social stigma and fear. Thus, the social, economic and physical aspects of the experience are closely inter-related.

In both developing and developed countries, awareness of the importance of a gender analysis in health is growing, with respect to both infectious and chronic diseases. Despite a rapidly-expanding literature in this area, comprehensive, integrative analyses are few. It is difficult to compare the many studies in this field as they are based on populations with different ethnic, socioeconomic and demographic characteristics, different geographic and ethnic groups, and on different diseases and health conditions, or different symptoms of these diseases and conditions. Moreover, these interrelationships may change over time, with, for example, changes in marital status, age, or changes in social and economic conditions. As a result, in-depth gender analyses of health and illness are very few. If gender studies are to provide a useful basis for the development of policy, planning, and health services, a more systematic approach to studies in this area are needed. Frameworks such as the one used in this paper are a useful beginning.

Women's health programmes, and increasingly gender studies programmes, are being incorporated into university health curricula. Nonetheless, such programmes are still mainly pursued by social scientists and are not seen as a mandatory part of biomedical training. Mainstreaming gender studies into biomedical programmes can greatly enhance awareness of a wider range health issues, thereby contributing to the prevention of illness and the mitigation of negative health outcomes. It can also stimulate much needed research on gender differences between developing countries and developed countries and on the impact of gender on the epidemiological transition from infectious to non-communicable diseases.
